# The histone‐like protein HupB influences biofilm formation and virulence in *Xanthomonas citri *ssp. *citri* through the regulation of flagellar biosynthesis

**DOI:** 10.1111/mpp.12777

**Published:** 2019-01-11

**Authors:** Valeria P. Conforte, Florencia Malamud, Pablo M. Yaryura, Laila Toum Terrones, Pablo S. Torres, Verónica De Pino, Cristian N. Chazarreta, Gustavo E. Gudesblat, Atilio P. Castagnaro, María R. Marano, Adrian A. Vojnov

**Affiliations:** ^1^ Instituto de Ciencia y Tecnología Dr. César Milstein, Fundación Pablo Cassará, CONICET Saladillo 2468 Ciudad de Buenos Aires C1440FFX Argentina; ^2^ Instituto de Investigaciones Biotecnológicas, Universidad Nacional de San Martín Campus Migueletes, 25 de Mayo y Francia General San Martín B1650HMN Provincia de Buenos Aires Argentina; ^3^ Centro de Investigaciones y Transferencia de Villa María CONICET Universidad de Villa María Carlos Pellegrini 211 Villa María, X5900FSE Córdoba Argentina; ^4^ Departamento de Fisiología Biología Molecular y Celular, Instituto de Biodiversidad y Biología Experimental y Aplicada, CONICET, Facultad de Ciencias Exactas y Naturales, Universidad de Buenos Aires Intendente Güiraldes 2160 Buenos Aires C1428EGA Argentina; ^5^ Instituto de Tecnología Agroindustrial del Noroeste Argentino (ITANOA), Estación Experimental Agroindustrial Obispo Colombres (EEAOC), Consejo Nacional de Investigaciones Científicas y Técnicas (CONICET) Av. William Cross 3150 Las Talitas C.P. T4101XAC Tucumán Argentina; ^6^ Instituto de Biología Molecular y Celular de Rosario, Departamento de Microbiología, Facultad de Ciencias, Bioquímicas y Farmacéuticas Universidad Nacional de Rosario Suipacha 531 Rosario S2002LRK Santa Fé Argentina

**Keywords:** biofilm, citrus, flagella, histone‐like protein, HU, pathogenicity, *Xanthomonas*

## Abstract

Citrus canker is an important disease of citrus, whose causal agent is the bacterium *Xanthomonas citri *ssp. *citri* (*Xcc*). In previous studies, we found a group of *Xcc *mutants, generated by the insertion of the Tn*5* transposon, which showed impaired ability to attach to an abiotic substrate. One of these mutants carries the Tn*5* insertion in *hupB*, a gene encoding a bacterial histone‐like protein, homologue to the β‐subunit of the Heat‐Unstable (HU) nucleoid protein of *Escherichia coli*. These types of protein are necessary to maintain the bacterial nucleoid organization and the global regulation of gene expression. Here, we characterized the influence of the mutation in *hupB* regarding *Xcc* biofilm formation and virulence. The mutant strain *hupB* was incapable of swimming in soft agar, whereas its complemented strain partially recovered this phenotype. Electron microscope imaging revealed that impaired motility of *hupB* was a consequence of the absence of the flagellum. Comparison of the expression of flagellar genes between the wild‐type strain and *hupB* showed that the mutant exhibited decreased expression of *fliC* (encoding flagellin). The *hupB* mutant also displayed reduced virulence compared with the wild‐type strain when they were used to infect *Citrus lemon* plants using different infection methods. Our results therefore show that the histone‐like protein HupB plays an essential role in the pathogenesis of *Xcc* through the regulation of biofilm formation and biosynthesis of the flagellum.

## Introduction

Citrus canker, one of the most important diseases of citrus trees, leads to defoliation, dieback and fruit drop, reducing yields and causing serious economic losses (Graham *et al.*, 2004). Its causative agent is *Xanthomonas citri *ssp. *citri *(hereafter *Xcc*), a member of the Gamma subdivision of Gram‐negative proteobacteria (da Silva *et al.*, 2002). This pathogen enters the host plant tissues through stomata or wounds, and then colonizes the apoplast, causing corky lesions (cankers) in fruits, leaves and stems. It finally breaks the tissues as a consequence of cell hyperplasia, hence allowing bacterial dispersal (Brunings and Gabriel, 2003). The canker itself, together with surviving populations of bacteria, constitutes the main source of disease spread (Cubero and Graham, 2004). Given the relevance of citrus canker, it is essential to undertake studies that expand the understanding of *Xcc *virulence mechanisms in order to improve its detection and control.

The successful infection of bacteria depends on the ability of the microorganism to adhere to the plant surface, invade the intracellular spaces of the host plant, obtain nutrients and overcome plant defence (Büttner and Bonas, 2010). Like other plant pathogens, *Xanthomonas *spp. display an array of virulence factors, such as the synthesis of extracellular cell wall‐degrading enzymes and the production of xanthan, the characteristic extracellular polysaccharide (EPS) of this genus, which contribute to the interaction with plants (Tang *et al.*, 1991; Vojnov *et al.*, 1998). The production of these factors is under the control of the *rpf*/DSF (diffusible signal factor) cell‐to‐cell chemical communication system (Barber *et al.*, 1997; Crossman and Dow, 2004; Dow *et al.*, 2003; Siciliano *et al.*, 2006; Tang *et al.*, 1991; Torres *et al.*, 2007).

Biofilm formation plays a key role in the induction of disease symptoms during *Xcc* infection. Biofilms are complex assemblies of bacteria usually attached to a solid surface (abiotic or biotic) and encapsulated in a matrix consisting mainly of EPS, proteins, extracellular DNA and lipids (Flemming and Wingender, 2010). In phytopathogenic bacteria, biofilm development contributes to the maintenance of a critical mass of bacteria in specific areas, which facilitates the infection process. Moreover, these bacterial assemblages confer resistance to harsh environmental conditions, protection from protozoa attack, tolerance to antimicrobial agents and consortia metabolism, and also enable horizontal gene transfer (Bogino *et al.*, 2013; Danhorn and Fuqua, 2007). Biofilm formation, which has been extensively studied in many species, consists of five general stages: an initial and reversible attachment, followed by an irreversible attachment that leads to the formation of a monolayer, the development of microcolonies that mature into complex macrocolonies (with the characteristic mushroom shape) and the dispersal of cells in the planktonic state (Kierek‐Pearson and Karatan, 2005; Martínez and Vadyvaloo, 2014; Sauer *et al.*, 2002; Stoodley *et al.*, 2002).

The biofilm formation process in *Xcc* requires the EPS, xanthan, for the development of mature structures both *in vitro* and *in vivo* (Rigano *et al.*, 2007). In many species of Gram‐negative bacteria, flagella are essential during the early stages of biofilm formation (Guttenplan and Kearns, 2013; Haiko and Westerlund‐Wikström, 2013; Wood, 2013). In particular, in *Xcc*, it has been shown that flagella are also involved in structuring the mature biofilm with defined water channels and play a vital role in bacterial dispersal, and thus are required for the establishment of bacterial communities on the leaf surface (Malamud *et al.*, 2011).

To better understand the whole mechanism of biofilm formation, it is necessary to know the genetic determinants and underlying factors that regulate this process. To this end, *Xcc* mutant libraries have been screened in search of new genes involved in biofilm development. Using this tool, Li and Wang (2011) found 33 novel genes related to regulatory networks, EPS production and lipopolysaccharide synthesis, among others. In our laboratory, a library of *Xcc* mutants obtained by the insertion of the Tn*5* transposon was screened in search of those affected in the adhesion to an abiotic substrate (Malamud *et al.*, 2013). As a result, we found 23 novel genes associated with biofilm formation in *Xanthomonas *spp., four related to the regulation of gene expression, five encoding membrane proteins, eight encoding structural proteins and six encoding hypothetical proteins. From this screening, we selected the strain characterized in this work, affected in the expression of *hupB* (XAC1081).

The XAC1081 gene, *hupB*, encodes a bacterial histone‐like protein, homologous to the β‐subunit of the Heat‐Unstable (HU) nucleoid protein (da Silva *et al.*, 2002). Histone‐like proteins in bacteria are able to bind to DNA and are known to contribute to the organization of the bacterial ‘nucleoid’ (Rouvière‐Yaniv and Gros, 1975). In addition, they are associated with cellular processes, such as replication, recombination and DNA repair, and the global regulation of gene expression (Dorman and Deighan, 2003). Histone‐like proteins are divided into four major groups based on their amino acid sequence: histone‐type *Escherichia coli* U93 (HU), nucleoid structuring histone (H‐NS), integration host factor (IHF) and factor for inversion stimulation (FIS) (Anuchin *et al.*, 2011). HU and H‐NS are the best characterized. HU does not recognize a specific sequence when binding to DNA, but prefers regions with distorted or supercoiled structures (Dorman and Deighan, 2003). It is believed that the functional role of HU is to participate in DNA supercoiling (Tanaka *et al.*, 1995). In *E. coli*, HU consists of two subunits, *hupA* and *hupB*, acting as an *hupA–hupA* homodimer or as an *hupA–hupB* heterodimer, depending on the growth phase of the bacterium (Balandina *et al.*, 2001; Grove, 2011). In most bacteria, HU is a homodimer. In *E. coli*, HU null mutants have a mild phenotype, whereas in Gram‐positive bacteria, such as *Bacillus subtilis*, HU appears to be essential (Grove, 2011). In addition, it has been described that HU participates in the regulation of genes involved in processes related to virulence (EPS production, mobility and expression of virulence factors) and tolerance to different types of stress (anaerobiosis, medium acidification, osmolarity increase, UV radiation, etc.) (Balandina *et al.*, 2001; Nishida *et al.*, 1997; Oberto *et al.*, 2009).

The main objective of this work was to analyse the role of an *hupB* homologue gene in *Xcc* biofilm formation and virulence. We found that a null mutation in the histone‐like protein HupB results in a reduction in the ability of the bacterium to infect *Citrus*. In addition, the *hupB* null mutant is impaired in flagellum synthesis, being unable to develop a mature biofilm structure. Here, we provide new insights into gene regulation by studying, for the first time, the role of a histone‐like protein in *Xcc*.

## Results

### The *hupB* gene encodes a bacterial histone‐like protein

**Figure 1:**
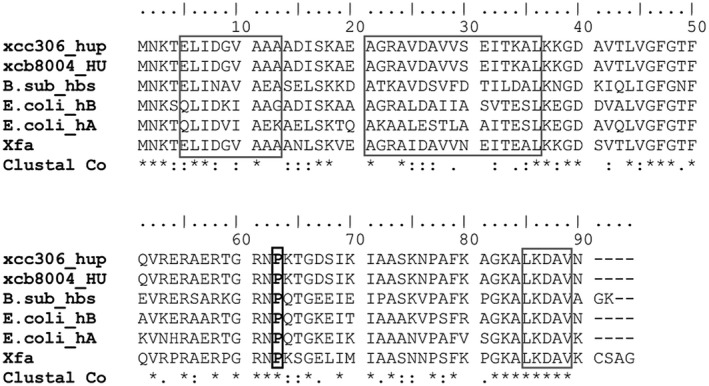
Multiple alignment of the HupB protein in *Xanthomonas citri *ssp. *citri* (*Xcc*) and its homologues in other species. Alignments were carried out using Clustal IX 2.1 software. Identical residues are indicated with an asterisk (*); highly conserved residues (:) and less conserved residues (.). Grey boxes indicate the sequences of the three α‐helix structures. The proline residue (that intercalates into DNA) is shown in a black box. References and National Center for Biotechnology Information (NCBI) protein IDs: xcc306_hup, *X. citri *ssp.* citri* strain 306 (AAM35959); xcb8004_HU, *X. campestris* pv. *campestris* strain 8004 (AAY50306); B.sub_hbs, *Bacillus subtilis* strain 168 (NP_380160); *E.coli*_hB, *hupB* of *Escherichia coli* strain K‐12 MG1655 (NP_414974); *E.coli*_hA, *hupA* of *E. coli* strain K‐12 MG1655 (NP_418428); Xfa, *Xylella fastidiosa* strain 9a5c (AAF84000).

The null mutant in *hupB* was isolated from a screening of a pool of *Xcc* 306 mutants (carrying the insertion of Tn*5*) that aimed to identify the genes required for cell attachment to an abiotic substrate (Malamud *et al.*, 2013). The *hupB* gene encodes a histone‐like protein homologous to the β‐subunit of the HU protein in *E. coli*. The predicted amino acid sequence shows that, as expected, HupB is a small protein with a predicted molecular weight of approximately 9 kDa. Protein sequence analysis reveals a high conservation among orthologous genes, not only within the same genus (*X. campestris* pv. *campestris*) or related species (*Xylella fastidiosa*), but also in bacteria evolutionarily more distant, such as *E. coli* or *B. subtilis* (Gram‐positive species) (Fig. 1).

Typically, HU adopts a compact conformation by linking the two monomers together (Swinger *et al.*, 2003). Two α‐helices of each monomer form the body of the protein, which is confined by two β‐sheets that extend and ‘embrace’ the DNA. An α‐helix (short sequence) completes the structure. According to Lee *et al. *(1992), a proline residue (P63) is essential in the ‘coupling’ with DNA, and its substitution results in a significant alteration in DNA binding. Another important residue is lysine 3 (K3), which is critical for maintaining a stable complex (Grove and Saavedra, 2002). All of these elements appear to be conserved in the protein encoded by the *hupB* gene (Fig. 1).

### A null mutation in* hupB* impacts negatively on the biofilm formation process

Biofilm formation is a dynamic process which is not only influenced by multiple factors, but also involves several stages, from adhesion to the surface until the appearance of mushroom‐like arrangements. One of our aims was to evaluate biofilm development in the *hupB* mutant in comparison with the wild‐type strain.

**Figure 2:**
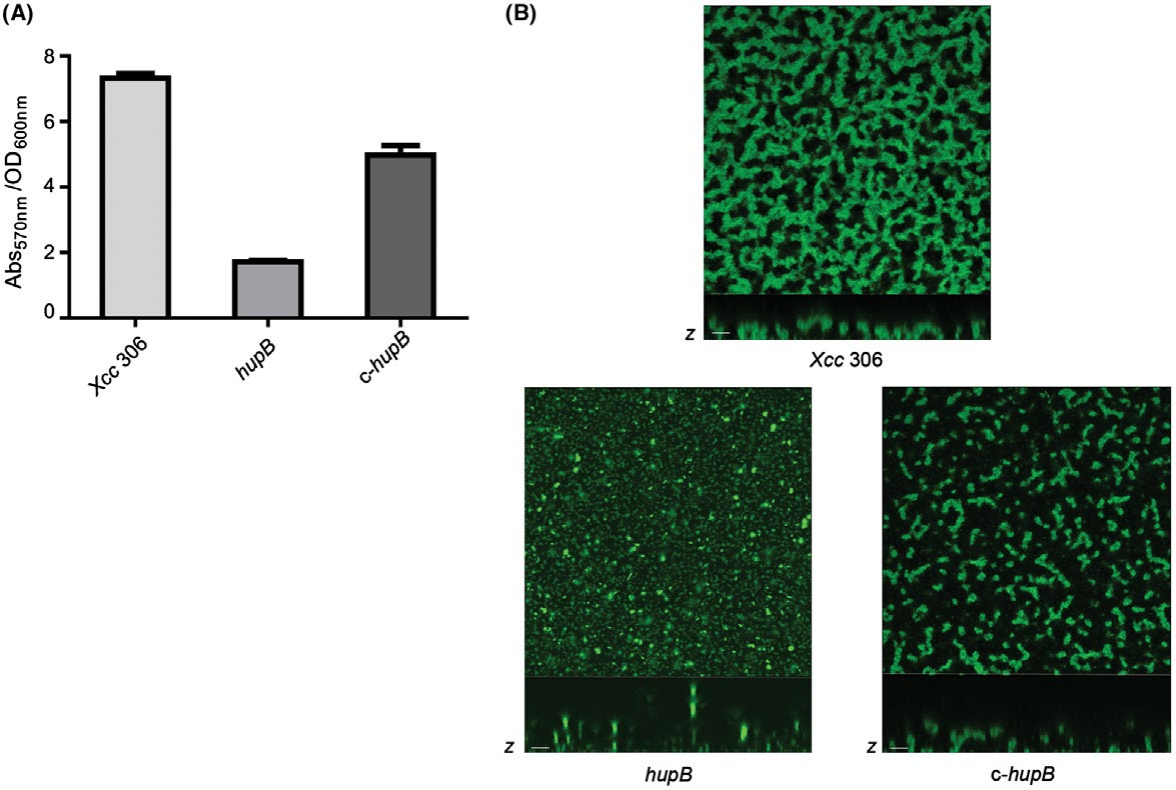
Biofilm formation analysis of the *Xanthomonas citri *ssp. *citri* (*Xcc*)* hupB* mutant strain. (A) Microtitre plate adhesion assay. Crystal violet absorbance (Abs_570 nm_) to OD_600_ ratio is represented for each strain: wild‐type strain (*Xcc* 306), null mutant strain (*hupB*) and the complemented strain (c‐*hupB*). Error bars indicate standard error of the mean of three independent experiments. (B) Biofilm structures after 4 days of inoculation: projections in the *x*–*y* plane were obtained through confocal laser scanning microscopy (CLSM) (40× magnification); *z*‐axis‐projected images are shown below (scale bars, 5 μm).

In the first instance, we confirmed that there were no major differences in growth between the *hupB* mutant and the wild‐type strain (Fig. S1, see Supporting Information). Then, we analysed the ability of each strain to attach to an abiotic surface, showing a significant reduction for the *hupB* mutant (approximately 60% less than the wild‐type strain) (Fig. 2A). The structural organization of the biofilm was studied in detail by confocal laser scanning microscopy (CLSM). The presence of a mature biofilm formed by the wild‐type strain was clearly observed after 4 days of incubation (Fig. 2B). In contrast, the *hupB* mutant strain was only able to grow into seemingly a monolayer of cells, very different from a typical biofilm (Fig. 2B). Complementation with an intact copy of the *hupB* gene and its promoter region (c‐*hupB*) partially restored the defective phenotypes (Fig. 2).

### The absence of HupB affects *Xcc* motility

**Figure 3:**
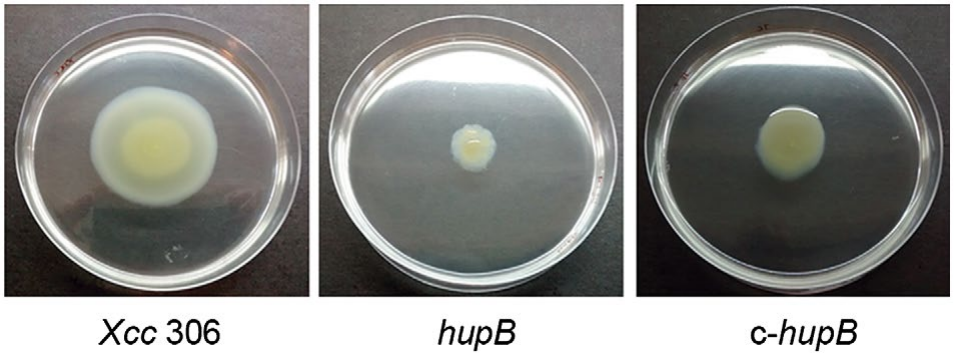
Swimming motility assay. Aliquots (3 µL) of each culture (*Xcc* 306, *hupB* and c‐*hupB*) in the exponential growth phase were placed in the centre of a Petri dish containing NYGB‐soft agar medium. The plates were incubated at 28 °C for 72 h, and photographs were taken. *Xcc*, *Xanthomonas citri *ssp. *citri*.

The environmental survival and pathogenesis of *Xcc* are subject to its ability to move. In addition, motility is one of the determinants of biofilm formation. Therefore, we investigated whether the absence of HupB affects bacterium motility. To this end, we performed a swimming assay in which soft agar plates were inoculated with aliquots of exponential phase cultures of the different strains. After 72 h of incubation, the *hupB* mutant was essentially non‐motile, whereas the wild‐type strain showed a migration area around the plating site (Fig. 3). Complementation of the *hupB* mutant strain restored the ability to swim, although not reaching the levels of the wild‐type strain.

### The absence of HupB leads to the loss of flagellum

**Figure 4:**
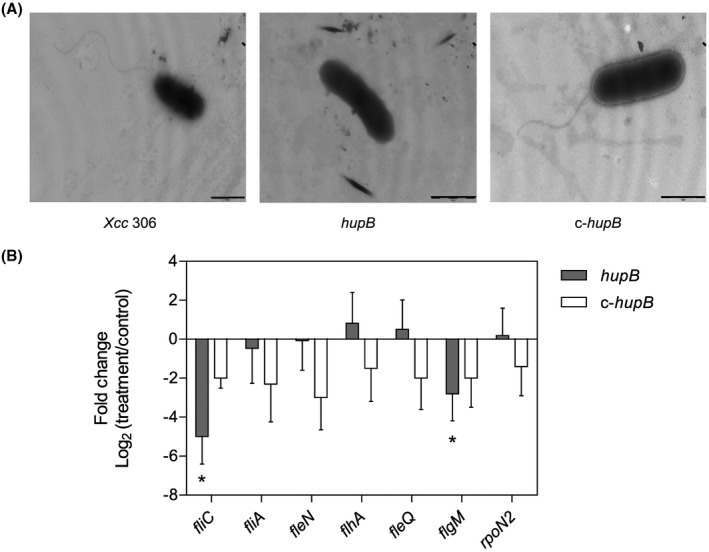
Study of the flagellar structure in the *hupB* null mutant. (A) Images obtained by transmission electron microscopy of the flagellar structure of the wild‐type strain *Xcc* 306, *hupB* mutant strain and the c‐*hupB* strain. Bacteria were cultured in rich medium and harvested when the optical density at 600 nm (OD_600_) = 1. Scale bar, 0.5 μm. (B) The relative expression of flagellar genes (*fliC*, *fliA*, *fleN*, *flhA*, *fleQ*, *flgM*, *rpoN2*) between the treatment (*hupB* mutant strain or the c‐*hupB* strain) and the control (wild‐type strain *Xcc* 306) was estimated by quantitative reverse transcription‐polymerase chain reaction (qRT‐PCR). Data correspond to the average of four independent experiments; error bars indicate standard error. Significant difference between treatment and control is indicated by an asterisk (*P* < 0.05). *Xcc*, *Xanthomonas citri *ssp. *citri*.

As the *hupB* mutant strain was non‐motile, we hypothesized that this could be associated with a functional or structural defect in the flagellum. Therefore, we examined the flagellar apparatus of exponentially growing cells by transmission electron microscopy (TEM). As shown in Fig. 4A, a single polar flagellum was visible in the wild‐type and in *c‐hupB*, but not in most of the observed fields for the *hupB* mutant strain. These results suggest a possible role of HupB in the regulation of flagellar biosynthesis.

Flagellum‐dependent motility in *Xanthomonas* spp. is controlled by the hierarchical expression of a regulatory cascade involving several regulators (Yang *et al.*, 2009). Thus, we compared the expression of some of these genes between the wild‐type strain and the mutant strain by quantitative reverse transcription‐polymerase chain reaction (qRT‐PCR) (Fig. 4B). According to our results, *fliC*, a class III gene encoding flagellin, was significantly reduced in comparison with the wild‐type strain. Genes encoding FleQ and RpoN2, class I master regulators that control the expression of class II genes, showed no significant differences between strains. By contrast, the *flgM* gene, encoding a class I master regulator, showed a significant reduction in its expression in the mutant strain when compared with the wild‐type strain. The levels of expression of the class II genes (*fliA*, *flhA* and *fleN*) displayed no differences between strains. Complementation with an intact copy of the *hupB* gene and its promoter region (c‐*hupB*) restored the expression of *fliC* and *flgM* genes to levels similar to those in the wild‐type strain (Fig. 4B).

### HupB affects *Xcc* pathogenicity

**Figure 5:**
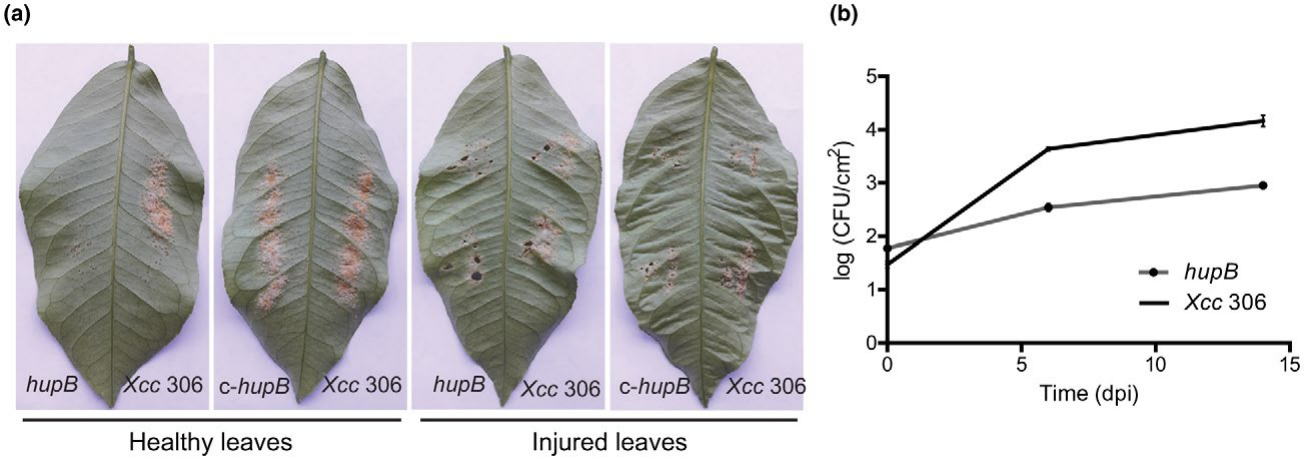
Pathogenicity assay in *Citrus limon* plants. (A) Development of disease symptoms in lemon leaves. Left: intact leaves were infected by swabbing with bacterial suspensions of the wild‐type strain (*Xcc* 306), the *hupB* null mutant (*hupB*) or the complemented strain (c‐*hupB*) at a final concentration of 1 × 10^8^ colony‐forming units (CFU)/mL; right, leaves were injured before swabbing. Photographs were taken at 30 days post‐infection (dpi). (B) *In vivo* growth of the *Xanthomonas citri *ssp. *citri* (*Xcc*) strains in lemon leaves. Bacterial suspensions (1 × 10^6^ CFU/mL) were swabbed onto previously injured leaves. Bacterial counts were determined at 0, 6 and 14 dpi. Three independent experiments were carried out and similar results were observed.

To study the possible effect of the *hupB* gene product in *Xcc* virulence, we carried out pathogenicity assays in lemon plants (*Citrus limon* cv. *genova*). Two different types of infection method were applied (Fig. 5A): swabbing bacterial suspensions on the lower surface (abaxial) of healthy young leaves (i), or on leaves previously injured with a needle to allow bacteria to enter the mesophyll (ii). Symptoms were evaluated at 30 days post‐infection (dpi). The results showed that, regardless of the infection method used, the null *hupB* mutant strain generated fewer lesions than the wild‐type strain (Fig. 5A). By contrast, complementation by the expression of the *hupB* gene under its own promoter (c‐*hupB*) restored the ability to induce cankers to wild‐type levels.

Next, we compared bacterial growth *in planta* (Fig. 5B). For this purpose, injured young leaves were swabbed with bacterial suspensions of the *hupB* mutant strain or the wild‐type strain. At 0, 6 and 14 dpi, plant tissue was ground and the number of colony‐forming units (CFU) per square centimetre of leaf tissue was determined. The results in Fig. 5B show significant differences between strains at 6 dpi. At 14 dpi, the wild‐type strain was able to grow about two‐fold more than the *hupB* mutant strain.

## Discussion

The *hupB* gene is homologous to the β‐subunit of the HU protein of *E. coli*. This histone‐like protein is a member of family II of the DNA‐binding proteins (DNABII), a group of small proteins involved in the organization and compaction of bacterial DNA (Dillon and Dorman, 2010). HU and other histone‐like proteins in bacteria are now called nucleoid‐associated proteins (NAPs). NAPs have been related to the formation of complexes at specific DNA structures, in the processes of recombination, initiation of replication and regulation of gene expression, in addition to their role in DNA compaction and protection. In general, bacteria present multiple NAPs, whose functions usually overlap and are complementary. For example, in *E. coli*, the lack of HU is not lethal, unless other NAPs, such as IHF and H‐NS, are also suppressed (Kayoko *et al.*, 1992). In other bacteria, HU disruption is lethal, as they only have one NAP available (Liu *et al.*, 2008; Micka and Marahiel, 1992). In the case of *Xcc*, there are genes in its genome that encode proteins homologous to IHF and H‐NS (da Silva *et al.*, 2002). In agreement with this, our results showed that a null mutation in *hupB* was not lethal.

In this work, we have attempted to understand why a null mutation in a histone‐like protein results in a reduction in the ability of the bacterium to adhere to a surface. To this end, we analysed structures which could be involved in biofilm formation and found that the mutant in *hupB* does not have a flagellum. However, we cannot reject other defective phenotypes which may contribute to biofilm formation. Notably, Devaraj *et al. *(2015) have recently described a new role of NAPs in biofilm formation in *E. coli*. These authors proposed that *hupB* may play a structural role during biofilm formation as an essential element of the matrix, and that HU, as a DNABII protein, binds to the extracellular DNA of the matrix. These authors also described that the HupB subunit is a necessary and limiting component for the growth and maintenance of biofilm structures.

In the present study, the *hupB* mutant strain was unable to swim because of the absence of a flagellum, which was associated with a significant reduction in the expression of *fliC* (a gene encoding the main protein of the filament). This suggests that *hupB* positively regulates flagellar synthesis in *Xcc*. Similar results have already been reported in *E. coli* by Nishida *et al. *(1997). The double mutant *hupA‐hupB* (i.e. mutant in both subunits of the protein) does not produce flagellin and, as a consequence, is non‐motile. Nishida *et al. *(1997) also concluded that the regulation of flagellar gene expression occurs at a transcriptional level. However, the mechanism by which *hupB* regulates *fliC* expression and motility has not been elucidated. Our results indicate that none of the class II or class I genes, with the exception of *flgM*, show differences in expression between the strains. Compared with the class I and II genes, *fliC* shows reduced expression in the mutant strain (Fig. 4B). From studies derived from the analysis of the *gal* operon of *E. coli*, it is presumed that HU could induce DNA looping, thus helping to load or unload negative or positive regulators in specific sites of DNA to help or block the start of gene transcription (Lewis *et al.*, 1999; Oberto *et al.*, 2009). We suggest that the absence of HU would alter the activity of the *fliC* promoter directly, causing the loss of accessibility for positive regulators or facilitating the access for negative regulators. HU promotes the compaction of DNA, and so its inactivation would alter the structure of the DNA molecule and, as a consequence, the activity of several promoters would be affected. Considering this hypothesis, i.e. that the *hupB* gene product affects the supercoiling of DNA (Nishida *et al.*, 1997), its mutation could alter the transcription of other genes that we have not considered in our analysis, but that are involved in flagellar motility. Nevertheless, the mechanism of synthesis and rotation of the flagellum is complex and involves many factors. HupB could alter motility in many ways, as reported for H‐NS in *E. coli*. On the one hand, H‐NS is a positive regulator of the flagellar regulon, binding DNA and promoting the synthesis of the master regulator FlhDC (class I) (Bertin *et al.*, 1994), whereas, on the other, H‐NS modulates flagella rotation by interacting with FliG, promoting its interaction with Mot proteins (flagellar motor).

In the present study, we found that inactivation of *hupB* leads to a reduction in bacterial virulence in lemon plants. Swabbing infection on undamaged leaves showed that the *hupB* mutant strain was less effective in colonizing plant tissue through natural openings, such as stomata (Fig. 5A). These observations probably imply that a lack of motility impairs bacterial movement on the leaf surface and, as a consequence, limits penetration through stomatal pores. Consistently, the absence of flagellin has been shown to reduce slightly *Xcc* pathogenicity in lemon leaves (Malamud *et al.*, 2011).

In this work, we focused on the study of the effect of the mutation in the *hupB* gene (XAC1081) on the biofilm formation and virulence of *Xcc*, and demonstrated that it is essential for both processes. However, more work is needed to understand more precisely how it regulates these functions.

## Experimental Procedures

### Bacterial strains and growth conditions

*Xcc *strains were cultured at 28 °C with shaking at 220 rpm in peptone–yeast extract–malt extract (PYM) medium (Cadmus *et al.*, 1976) or Y minimal medium (YMM) (Sherwood, 1970). *Escherichia coli *strains were grown at 37 °C in Luria–Bertani (LB) medium (Sambrook and Russell, 2001). Bacterial growth was measured in a T60UV‐Visble spectrophotometer (PG Instruments, Lutterworth, Leicestershire, UK) at 600 nm, and described as the optical density at 600 nm (OD_600_). When required, antibiotics were added to the growth media in the following concentrations: ampicillin (200 mg/mL), kanamycin (50 mg/mL) and tetracycline (3.5 mg/mL).

### Complementation of the *Xcc hupB* mutant strain

The null mutant *hupB* was complemented through the introduction of the pLAFR3 plasmid carrying an intact copy of the *hupB* gene and its promoter region. The *hupB* gene and its promoter were amplified using the primers BamHI‐*hupB*‐rv and EcoRI‐*hupB*‐fw (Table S1, see Supporting Information). The PCR product was cloned into the pGEMTeasy vector (Promega, Madison, WI, USA) and then digested with *Eco*RI. The released fragment was ligated into pLAFR3 (Staskawicz *et al.*, 1984), which had been digested previously with the same restriction enzyme. This construct was introduced into the *hupB* mutant strain by biparental conjugation through *E. coli* S17.

### Biofilm formation analysis

#### Adhesion assay

To measure the attachment of bacteria to an abiotic surface, we performed the crystal violet assay described by O’Toole and Kolter (1998). Briefly, bacterial strains cultured overnight in PYM medium were used to inoculate YMM (containing 1% w/v of glucose as the sole carbon source) to a final OD_600_ of 0.1. Aliquots of 150 μL of these suspensions were placed in different wells of a polystyrene microtitre plate (Orange Scientific, Braine‐l’Alleud, Belgium) and incubated at 28 °C for 24 h without shaking. Differences in growth between strains were rejected by determining the OD_600 _of each strain before performing the assay. Initially, the medium was carefully removed from each well with a pipette and the wells were then washed with NaCl (0.9% w/v). Then, cells bound to the wells were stained with a 0.1% (w/v) crystal violet solution. After 30 min of incubation, the remaining crystal violet solution was removed and the wells were washed twice with distilled water. The crystal violet adhering to each well was dissolved in 150 μL of 70% (v/v) ethanol. The absorbance of each well was measured with a 7520 Microplate Reader (Cambridge Technology Inc., Bedford, MA, USA) at 570 nm. The results are expressed as the ratio between crystal violet absorbance at 570 nm and growth (OD_600_).

#### *In vitro* analysis of biofilm formation by CLSM

All strains were cultured in PYM medium (supplemented with the corresponding antibiotic) at 28 °C. Cultures were diluted 1 : 1000 in YMM, and aliquots of 500 μL were transferred to chambered coverglass slides containing a 1‐mm‐thick borosilicate glass (no. 155411) (Thermo Scientific Nunc Lab‐Tek, Waltham, MA, USA). Bacterial suspensions were cultured in the chambers for 4 days at 28 °C without shaking until analysis (Malamud *et al.*, 2011). To visualize samples, bacteria were stained using the BacLight LIVE/DEAD viability kit (Thermo Fisher Scientific, Waltham, MA, USA). This kit consists of two different fluorescent dyes: Syto‐9t (S‐9) (highlighting viable bacteria) and propidium iodide (indicating dead cells). Biofilm formation was studied through an inverted confocal laser scanning microscope (Nikon Eclipse TE 2000‐E2, Nikon, Melville, NY, USA). Three‐dimensional images were generated with Image J 1.49 software from the National Institutes of Health (http://rsbweb.nih.gov/ij/download.html).

### Swimming motility assays

Swimming assays in soft agar were carried out as described by Malamud *et al. *(2011). Bacteria were cultured in PYM medium overnight and an aliquot (3 μL) of each strain was placed in the centre of a Petri dish containing NYGB medium [0.5% (w/v) peptone extract, 0.3% (w/v) yeast extract and 16 mL/L glycerol; 0.25% (w/v) agar]. The growth of each culture was normalized by OD_600_. Plates were incubated at 28 °C and images were taken 72 h later.

### Study of bacterial flagella by electron microscopy

To obtain TEM images, Formvar‐coated copper grids were floated on a drop (5–10 μL) of appropriately diluted bacteria for 1 min. Then, the grids plus absorbed bacteria were rinsed rapidly with distilled water and floated on a solution of uranyl (2%) for 30 s. Then, the grids were removed with forceps, rinsed with distilled water and the excess liquid was drained off with the edge of a filter paper and preparations were air dried for 5 min. Finally, the specimens were examined with a Zeiss LEO906 TEM (Carl Zeiss, Oberkochen, Germany) (operated at an accelerating voltage of 100 kV) and photographed with a Megaview III camera (Olympus, Center Valley, PA, USA).

### Gene expression analysis by RNA extraction, cDNA synthesis and qRT‐PCR

*Xcc *strains were cultured in PYM medium until they reached an OD_600_ between 1 and 1.5. Bacteria were harvested and total RNA was extracted by treatment with TRIzol (Thermo Fisher Scientific), following the manufacturer’s instructions. Total RNA was quantified by spectrometry and its integrity was checked by agarose gel run.

Total RNA was reverse transcribed using random primers and M‐MLV RT (Promega). All primers used in this work (Table S1) were designed with the software Primer Express 3.0 (Applied Biosystems, Foster City, CA, USA). Reactions were performed using SybrGreen master mix (Roche, Mannheim, Germany) and a Step One Real Time‐PCR system (Applied Biosystems), as described previously (Yaryura *et al.*, 2015). The protocol for the qRT‐PCRs was as follows: 50 °C for 2 min, initial denaturation at 95 °C for 5 min, followed by 40 cycles of 10 s at 95 °C and 30 s at 60 °C. qRT‐PCR data analysis and primer efficiencies were obtained using LinReg PCR software (Ramakers *et al.*, 2003). The 16S gene was used to standardize the expression of a given target gene; then a ratio between treatments was calculated using the algorithm developed by Pfaffl (2001). Relative expression ratios and statistical analyses were performed using the fgStatistics software interface (http://sites.google.com/site/fgStatistics/). The cut‐off for statistically significant differences was set as *P* < 0.05, indicated as *.

### Plant growth conditions and pathogenicity assays

The host plant in this work was *C. limon* cv. Genova. Plants were kept under controlled humidity and temperature (28–30 ºC) with a photoperiod of 12 h.

To carry out infection assays, bacteria were cultured in PYM medium supplemented with the appropriate antibiotic overnight. Aliquots of these cultures were diluted in distilled water to a final concentration of 1 × 10^6^ CFU/mL. These bacterial suspensions were swabbed onto the abaxial face of intact young leaves or previously injured leaves. Symptoms were observed at 30 dpi.

Bacterial growth *in planta* was quantified as described previously (Malamud *et al.*, 2012). Briefly, bacterial suspensions of known concentration (1 × 10^8^ CFU/mL) were swabbed on injured lemon leaves. Three samples were taken for each strain at 0, 6 and 14 dpi. Bacterial counts were determined by macerating 1‐cm^2^ leaf discs in 0.5 mL of sterile water. Suspensions were subjected to serial dilutions and cultured in medium‐rich plates with the corresponding antibiotic. Plates were incubated at 28 °C, and colonies were quantified after 48 h. Population data were transformed to log_10_ values, and standard errors were determined.

## Supporting information


**Fig. S1  **Growth curves of the strains under study in the different culture media: peptone–yeast extract–malt extract (PYM) (A) and Y minimal medium (YMM) (B).

**Table S1 **Strains, plasmids and primers used in this work.
