# Supplementary figures and images for: The histone‐like protein HupB influences biofilm formation and virulence in Xanthomonas citri ssp. citri through the regulation of flagellar biosynthesis
Source: Mol Plant Pathol. 2019 Jan 11;20(4):589–98. doi: 10.1111/mpp.12777 (PMC6637892; doi:10.1111/mpp.12777)

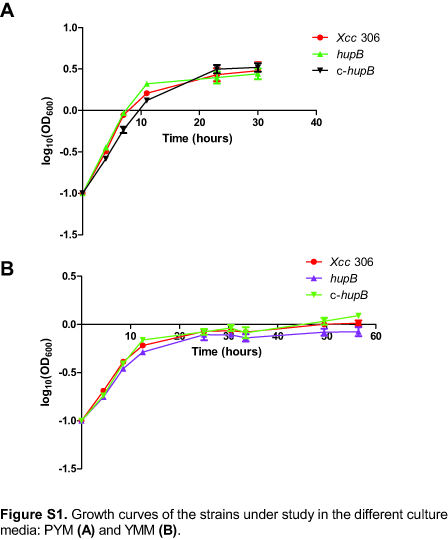

Supplement: Supplementary file 1 — Fig. S1 Growth curves of the strains under study in the different culture media: peptone–yeast extract–malt extract (PYM) (A) and Y minimal medium (YMM) (B). [file MPP-20-589-s001.tif]
