# Supplementary material for: The histone‐like protein HupB influences biofilm formation and virulence in Xanthomonas citri ssp. citri through the regulation of flagellar biosynthesis
Source: Mol Plant Pathol. 2019 Jan 11;20(4):589–98. doi: 10.1111/mpp.12777 (PMC6637892; doi:10.1111/mpp.12777)
Supplement: Supplementary file 2 — Table S1 Strains, plasmids and primers used in this work. [file MPP-20-589-s002.docx]

**Table S1.** Strains, plasmids and primers used in this work.

| **STRAINS** | **Relevant properties** | **Source** |
| --- | --- | --- |
| *Escherichia coli* DH5α | SupE44 Δ*lacU169* (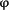 80*lacZ*ΔM15) *hsdR17 recA1 endA1 gyrA96 thi-1 relA1* | **(Hanahan, 1983)** |
| *E. coli* 2073 | DH5α carrying RK2073 | (Better and Helinski, 1983) |
| *Xanthomonas citri subsp.*  *citri* 306 (*Xcc* 306) | Wild type strain; Amp^r^ | (da Silva et al., 2002) |
| *Xcc hupB* | Xcc 306 carrying the Tn*5* insertion in XAC1081; Amp^r^ Km^r^ | (Malamud et al., 2013) |
| *Xcc* c-*hupB* | *Xcc hupB* carrying pLAFR3:*hupB*; Amp^r^ Km^r^ Tc^r^ | This work |
| **PLASMIDS** | **Relevant properties** | **Source** |
| pGEMT-easy | Vector; Amp^r^ | Promega |
| pLARF3 | Expession vector of *Xanthomonas* spp.; Tc^r^ | (Staskawicz et al., 1987) |
| **PRIMER** | **Sequence (5’-3’)** | **Usage** |
| BamHI-*hupB*-rv | ATGGATCCGGGAGACGCTCTACCGC | Cloning |
| BamHI-*hupB*-fw | ATGAATTCTCAAGCACTAACGAGGGC | Cloning |
| *16s* foward | CTGGAAAGTTCCGTGGATGTC | qRT-PCR |
| *16s* reverse | TGGTAGTCCACGCCCTAAACG | qRT-PCR |
| *fliC* foward | CAGCTTGGTGCCGTTGAAGTTG | qRT-PCR |
| *fliC* reverse | TGAACGCTCAGCGGAACCTCA | qRT-PCR |
| *fliA* foward | CCGCATCGCCCACCATCTG | qRT-PCR |
| *fliA* reverse | GCCCTGTTCGGAGTCGTAGC | qRT-PCR |
| *flgM* foward | CGACCGACAGCGTCAAGTTG | qRT-PCR |
| *flgM* reverse | GCCGAGGACTGCGAAAGC | qRT-PCR |
| *rpoN2 forward* | GAGACCGCCACCAACAAC | qRT-PCR |
| *rpoN2 reverse* | GTCCCACATCGTGCTTTCC | qRT-PCR |
| *fleQ forward* | TGCTGCTGGACGAAATTG | qRT-PCR |
| *fleQ reverse* | ACACATTGAGGCGATAGAAC | qRT-PCR |
| *flhF* forward | CCAGCAATTACGACGAAGAG | qRT-PCR |
| *flhF* reverse | GGCAGACGGTTGAGCAG | qRT-PCR |
| *flhA* forward | GCTGTTGATGACCACGATGC | qRT-PCR |
| *flhA* reverse | TGATGACCACGAAGTTGATGATG | qRT-PCR |
| *fleN* foward | AAGGGCGGCGTGGGGAAAAC | qRT-PCR |
| *fleN* reverse | GAGGTCGGCGTCAAGCAGCAG | qRT-PCR |

Amp^r^, Km^r^, Tc^r^ indicate ampicillin, kanamycin and tetracycline resistance, respectively.
